# Exploring alternative solvents to n-hexane for green extraction of lipid from camellia oil cakes^☆^

**DOI:** 10.1016/j.fochx.2025.102443

**Published:** 2025-04-05

**Authors:** Yingyi Lin, Yong Wang, Ying Li

**Affiliations:** a China-Malaysia Belt and Road Joint Laboratory on Oil Processing and Safety, Jinan University, Guangzhou 510632, China; b Department of Food Science and Engineering, Jinan University, Guangzhou 510632, China

**Keywords:** Alternative green solvents, Camellia seed oil cake, Lipid extraction, 2-Methyloxolane, Cyclopentyl methyl ether, Kinetics

## Abstract

*Camellia oleifera* Abel. is an important woody oilseed tree to help increase the self-sufficiency rate. In this study, lipid extraction focuses more on the solvent extraction of Camellia seed oil (CO) cakes rather than conventional cold pressing, where the performance of candidate alternative green solvents selected from hurdle technology was first evaluated. Compared to n-hexane (89.50 ± 0.00 %) and subcritical n-butane (83.75 ± 0.43 %), 2-methyloxolane (2-MeOx) performed the best with the comprehensive consideration of extraction ratio (94.79 ± 0.00 %), lipid composition and environmental impact (0.38 ± 0.07 kg of CO_2_ emission). Intriguingly, 2-MeOx exhibited the highest diffusion rate at both 25 °C and 55 °C in the extraction kinetic study, which could also extract the highest total phenolic contents (351.6 ± 0.02 mg GAE/kg dw) corresponding to its best oxidative stability. Besides, Hansen solubility modeling could help better understand the dissolving mechanism. 2-MeOx was demonstrated as the optimal alternative bio-based solvent to n-hexane with comparable extractability and selectivity in the CO extraction.

## Highlights

• Camellia oil could be efficiently extracted from its cake using green solvents.
• The effect of different solvents on the Camellia oil extraction was firstly studied.
• 2-MeOx performed the best among green solvents for Camellia oil extraction.
• The diffusion rate of green solvents was firstly studied in the kinetic study.
• The dissolving mechanism of solvents could be explained by theoretical miscibility.

## 1 Introduction

*Camellia oleifera* Abel. is one of the world's four major woody oilseed tree species, which is widely cultivated in subtropical regions of China and Southeast Asia (Ye et al., 2023). Since its cultivation has no competition with grain for arable land, it was listed as a national bulk oilseed crop in China in 2016. The production area of camellia seed in China is expected to reach 6 million hectares in 2025, indicating that a growing number of oil cakes will be generated from camellia seed pressing. These camellia oil cakes are normally treble the amount of camellia seed oil (CO) obtained (Xie et al., 2023). CO is known as the “oriental olive oil” due to its similar fatty acid composition to olive oil, especially for the high content of oleic acid (Dou et al., 2024). Numerous studies have demonstrated that CO is rich in oleic acid, squalene, phytosterols, polyphenols, and other bioactive compounds, which contribute to various health benefits including lipid regulation, liver protection, anti-asthmatic, anti-inflammatory and antioxidant activities (Li, Liu, et al., 2022; Li, Ma, et al., 2022).

Industrially, the shelled camellia seeds are usually employed to produce oil by mechanical expression, resulting in 5–10 % of residual oils in the CO cake (Qiu et al., 2024). Nevertheless, few companies use solvent extraction for improving CO yield due to the influence of solvent on the quality of CO. In general, n-hexane with low boiling point and good lipid dissolving capacity has been extensively used to extract hydrophobic compounds like lipids. However, such petroleum-based solvent is considered as the class 2 reproductive toxicity and class 2 aquatic chronic toxic solvent, which long-term exposure and use may cause human health and environmental problems (Chemat et al., 2019). Although some companies attempted to reuse CO cakes for solvent extraction like bulk oilseeds (e.g., soybean, rapeseed, etc.), the consumer acceptability of CO's naturalness and sensory quality is still the challenge for commercialization.

Due to the increasing environmental and safety concerns with the development of Green Chemistry, seeking alternative solvents to n-hexane has become a major issue for both academia and the industrial community. The alternative green solvent refers to a non-volatile renewable solvent that conforms to the concept of green chemistry, whose use can reduce harmful effects on both human health and the environment (Mostafazadeh et al., 2021). Green solvents are generally divided into five categories including water, bio-based solvents, supercritical carbon dioxide, liquefied gases, and natural deep eutectic solvents (Hessel et al., 2022), wherein the bio-based solvent has the advantages of availability, degradability, lower carbon footprint, and toxicity. Although subcritical fluid extraction has the advantages of energy saving, less solvent residue, and wider application (Ferreira et al., 2022), its frequently-used solvent n-butane still comes from nonrenewable petroleum sources, and its limited processing capacity cannot achieve large-scale continuous production. Hence, the selection of suitable alternative solvents makes it possible to extract target-specific molecules like lipids for future sustainable development (Gu & Jérôme, 2013). To avoid traditional time-consuming trial-and-error experiments, the hurdle concept was implemented for a rapid screening of alternative green solvent candidates. Hurdle technology refers to a comprehensive technical measure to control food safety by using the individual effect of a single hurdle factor or the interaction between multiple hurdle factors during food design and processing (Bigi et al., 2023). It is initially applied in various food processing sectors (e.g., fruits and vegetables, meat, milk and dairies, etc.) with the main objective of ensuring the microbial safety and extending the shelf life of food materials. However, a cost-effective hurdle approach has been attempted to prescreen solvents with appropriate hurdle factors (i.e., boiling point, Log *P* value, safety, etc.) selected corresponding to the ideal properties of green solvents (Janicka et al., 2022).

The objective of this work is to investigate an optimal alternative bio-based solvent to petroleum-based n-hexane for lipid extraction from camellia oil cake. In this study, alternative solvents including 2-methyloxolane (2-MeOx), cyclopentyl methyl ether (CPME), and ethyl acetate were first screened out from the GSK's solvent sustainability guide to replace n-hexane for lipid extraction from CO cakes. The effect of candidate alternative solvents was compared with n-hexane and subcritical n-butane as reference solvents in terms of acylglycerol and fatty acid composition, as well as lipid concomitants including tocopherols, sterols, squalene, and phenols. Furthermore, the Hansen solubility parameter (HSP) modeling was predicted to better understand the dissolving mechanism underlying such CO extraction. In addition, the kinetic model of lipid extraction from CO cakes was developed to further investigate the dissolving mechanism and the feasibility of alternative solvents for industrial-scale production with the consideration of energy consumption.

## 2 Materials and methods

### 2.1 Materials

Cold-pressed Camellia seed oil and cake were provided from Nanyuefuhe Eco-Agricultural Technology Co. Ltd. (Heyuan, China), where the oil cake was dried at 50 °C for 12 h, pulverized and sieved through a No.60 stainless mesh screen (0.25 mm). The resulting powders were used for the measurement of moisture and oil content, and the following solvent extractions.

2-MeOx, CPME and Folin-Ciocalteu reagent were purchased from Aladdin Biochemical Technology Co. Ltd. (Shanghai, China) while n-hexane, n-butane, ethyl acetate, ethanol, isopropanol, methanol, potassium hydroxide, trichloromethane were from Guangzhou Chemical Reagent Factory, China. Tocopherol, sterol and squalene standards were purchased from Sigma-Aldrich, Saint Louis, MO, USA. All solvents and chemicals used were of analytical grade.

### 2.2 Moisture and oil content measurement

The initial moisture content was measured using a SN-720 rapid moisture analyzer (Sanli Chemicals, Shenzhen, China). The oil content was evaluated by Soxhlet extraction. Camellia oil cake powders (3 g) wrapped in the filter paper were placed in a thimble, which was inserted into a Soxhlet apparatus and extracted with 100 mL of petroleum ether. The extraction was performed at 65 °C for 7 h and oils were collected after solvent evaporation. The oil content was calculated as follows,

(1) $$\text{Oil content}\;\left(\%\right)=\frac{m_{1}}{m_{2}}\times 100$$

where m_1_ is the weight of oil obtained after Soxhlet extraction (g), m_2_ is the weight of dry sample for extraction (g).

### 2.3 CO extraction

Camellia oil cake powders (30 g) were placed in a beaker and then stirred with the solvent at the solid: liquid ratio of (1:10, *w*/*v*) for 1.5 h at room temperature. After extraction, the solvent was removed by a rotary evaporator at 45 °C, and the process is repeated until the difference between the two consecutive weights is less than 10 % (*w*/w). Pilot-scale subcritical n-butane extraction was also conducted as the reference under the pressure of 0.55–0.60 MPa for 1 h, where the temperature was 65–70 °C and n-butane could be easily recovered by reducing pressure. The energy consumption was determined with a wattmeter at the power generator entrance and electrical heater power supply.

The oil extraction ratio was calculated as follows,

(2) $$\text{Oil extraction ratio}\;\left(\%\right)=\frac{m_{3}}{m_{2}\times \text{oil content}}\times 100$$

where m_3_ is the weight of oil obtained after solvent extraction (g).

### 2.4 Mass transfer kinetics

The solvent extraction process consists of a fast step (washing stage) and a slow step (diffusion stage). In the fast step, the oil attached to the surface of the particles is quickly washed in the extraction solvent. In the slow step, oil diffuses from the interior of the particles to the solvent depending on mass transfer, solid-liquid ratio, and particle size.

The modified model of Fick's Law of diffusion was used for CO extraction in the kinetics study (Kumar et al., 2023). The solution of Fick's equation is given as below,

(3) $$\frac{M_{t}}{M_{\infty }}=1-\sum_{n=1}^{\infty }A_{n}e^{-B_{n}t}\;$$

where t is the diffusion time (min) and M_t_ and M_∞_ represent the mass of the substance (kg oil/kg dry defatted meal) that diffused at time t (min) and infinite time, respectively.

Camellia seed cake powders suspended in a homogeneous medium are considered as spherical geometry with radius of 107.77 ± 1.69 μm in the proposed model, where the diffusion process takes place in a non-stationary state. The spherical geometry was measured by the laser diffraction particle size analyzer (Shimadzu SALD-2300, Japan) at 25 °C.

The initial time (*t* = 0) of rapid non-diffusion phenomenon was considered in the model, where the free surface oil (M_0_) of the meal was washed by fresh solvent. The conditions can be represented as:

(4) t=0,M=0

(5) $$t=t_{0},M=M_{0}\;$$

(6) $$t=t,M=M_{t}\;$$

(7) $$t\to t_{\infty },M=M_{\infty }$$

and the Fick's equation can be rewritten as

(8) $$\frac{M_{t}}{M_{\infty }}=1-\left(1-\frac{M_{0}}{M_{\infty }}\right)\sum_{n=1}^{\infty }A_{n}e^{-B_{n}\left(t-t_{0}\right)}$$

For sufficiently long times the Eq. (8) can be simplified to Eq. (9).

(9) $$\frac{M_{t}}{M_{\infty }}=1-Ae^{-\mathrm{Bt}}\;$$

where the coefficients A and B for spherical geometry can be showed as:

(10) $$A=\frac{6}{\pi^{2}}\;$$

(11) $$B=\frac{D_{e}\times \pi^{2}}{R^{2}}$$

where D_e_ represent the diffusion coefficients during the extraction process.

Once the optimized D_e_ value was obtained, the root mean square error (RMSE) was calculated as a statistical indicator using between actual and predicted data about lipid extraction ratio to test the predictive accuracy of the diffusion in the Eq. (12):

(12) $$\mathrm{RMSE}=\sqrt{\frac{1}{n}\sum_{i=1}^{n}{\left({\frac{M_{t}}{M_{\infty }}}_{\mathrm{act}}-{\frac{M_{t}}{M_{\infty }}}_{\mathrm{pre}}\right)}^{2}}\;$$

### 2.5 Determination of physicochemical properties

The physicochemical properties of extracted oil were measured according to AOCS official methods. Acid value (AV) and peroxide value (POV) of the extracted oils were measured according to the AOCS official methods AOCS official method Cd 3d-63 (2017), AOCS official method Cd 8b-90 (2017). For AV, the oil extracted (10 g) was dissolved in 100 mL toluene-isopropanol solution (1:1 *v*/v), where several drops of phenolphthalein were added. A standard solution of potassium hydroxide (0.1 M) was used for titration afterwards. The titration was determined to be completed when the color of the final solution turned light red and did not disappear in 15 s. For POV, the oil extracted (5 g) was dissolved in 30 mL of glacial acetic acid-chloroform solution (3:2 v/v), where 0.5 mL of saturated potassium iodide solution was added and held for 1 min. A standard solution of sodium thiosulphate solution (0.1 M) was used for titration after 30 mL of distilled water was added. When the yellow color of the solution disappeared, several drops of starch indicator were added and the titration started again. The titration was determined to be completed when the blue color of the final solution faded away and did not change in 15 s.

### 2.6 Acylglycerol and fatty acid compositions analysis

The composition of acylglycerols and fatty acids was analyzed by a gas chromatography (GC7820A, Agilent Technologies, Wilmington, DE, USA) equipped with a flame ionization detector (FID). For acylglycerol composition, oil samples dissolved in hexane at the concentration of 10.0 mg/mL were filtered through filter membranes of 0.45 μm. According to the AOCS official method Ce 5–86 (2017), A capillary DB-1ht column (15 m × 0.25 mm × 0.1 μm) was used with nitrogen as a carrier gas at a flow rate of 1 mL/min. Samples (0.5 μL) was injected with a split ratio of 20:1. The temperature of both detector and injector was 380 °C. The initial temperature of oven was set as 50 °C, which was heated to 100 °C at 50 °C/min, then increased to 220 °C at 80 °C/min and to 290 °C at 30 °C /min, respectively. This temperature rose up to 330 °C and to 380 °C at 50 °C/min and separately maintained for 2 min and 3 min.

According to AOCS official method Ce 2–66 (2017), the fatty acid composition in the oil sample was determined after transmethylation to fatty acid methyl esters (FAME). GC-FID was performed by a CP-sil88 capillary column (100 m × 0.25 mm × 0.2 mm) using nitrogen as the carrier gas at a flow rate at 28 mL/min. About 0.8 μL of sample was injected with a split ratio of 40:1. The temperature for both injector and detector was 260 °C. The oven temperature was programmed to maintain at 120 °C for 3 min, then heated to 175 °C at 8 °C/min and held for 18 min. All data were collected with Agilent EZChrom Elite software. The fatty acid composition was by comparison with 37 FAME standards (Supelco). Both acylglycerol and fatty acid composition were quantified as relative percentages of the total acylglycerols and fatty acids.

### 2.7 Analysis of lipid concomitants

#### 2.7.1 Tocopherol

The oil sample (3 g) was dissolved in n-hexane of HPLC grade (9 mL), which was then sonicated for 3 min before passing through a 0.22 μm polytetrafluoroethylene (PTFE) filter. Tocopherols were analyzed by an HPLC (Waters e 2695, Milford, MA) system equipped with a diode array detector at 292 nm using a Zorbax RX-Sil column (250.0 mm × 4.6 mm × 5.0 μm; Agilent, USA) at 40 °C. The solution of hexane: isopropanol (98.5:1.5, *v*/v) was prepared as the mobile phase in the HPLC system with the flow velocity of 0.8 mL/min.

#### 2.7.2 Phytosterol and squalene

The content of phytosterol and squalene was slightly modified according to the previous methods (Wang et al., 2021). Internal standard (5α-cholesterol) of 6 mg and 2 mL of 0.5 mol/L KOH-ethanol solution were added to the oil sample (800 mg). The mixed solution was shaken well and heated at 85 °C in the oven for 1 h. The mixture was cooled down and added with 1 mL of distilled water and 4 mL of n-hexane to extract unsaponifiable matter for 3 times. The extracts (3 mL) was then dried under nitrogen and silylated with 600 μL of derivatization reagent *N*,*O*-Bis(trimethylsilyl)trifluoroacetamide with trimethylchlorosilane (BSTFA+1 % TMCS) for 0.5 h at 75 °C. Finally, the cooled mixture was injected into the sample bottle (1 μL) through a PTFE filter (0.22 μm) for the following analysis with a split ratio of 100:1. A gas chromatograph-mass spectrometer (Agilent 7890B—7000C, USA) was equipped with a flame ionization detector using a DB-5 capillary column (0.25 μm, 30 m × 0.25 mm). The temperature for both flame ionization detector and injector was 280 °C, and the temperature of the ion source was set at 250 °C. Helium was used as the carrier gas at a flow rate at 1 mL/min. The temperature of oven was initially set at 200 °C for 1 min, which increased to 300 °C at 10 °C/min and held for 18 min. The ionization mode with electronic impact was used, which mass spectra were acquired over the mass range of 10–1000 u. MS Data collected were compared with their fragmentation patterns stored in the NIST14.L database.

#### 2.7.3 Total phenolic content (TPC)

The TPC in oil samples was quantified with slight modifications according to the previous method (Zhuang et al., 2018). The oil sample (1 g) was added with 1 mL of 80 % methanol in water (*v*/v) and 1 mL of n-hexane in tubes of 10 mL, which was centrifuged at 10000 rpm for 5 min after ultrasonication for 3 min. The supernatants were evaluated for the determination of TPC according to the Folin-Ciocalteu colorimetric assay, where the UV-VIS spectrophotometer was employed to measure the absorbance against the blank at 765 nm. The TPC measurements were performed in triplicate and TPC was given in mg of gallic acid equivalent per kilogram of dried weight (mg GAE/kg dw).

### 2.8 Oxidation stability analysis

The oxidation stability analysis was slightly modified according to the previous method (Naik et al., 2014). A differential scanning calorimeter (TA Instruments, New Castle, USA) was used for the determination of oxidation onset temperature (OOT) of oils extracted (Almoselhy, 2020). Oil samples (5–10 mg) were placed in 100 μL of open aluminum pans with an empty and the same size aluminum pan served as the reference. The flow velocity of the oxygen was controlled at 50 mL/min. Each oil sample was heated from 30 °C to 300 °C at a rate of 10 °C/min to obtain the OOT, which was analyzed in triplicate.

### 2.9 Solubility prediction methods

According to the classical “like dissolves like” rule, Hansen solubility parameters (HSP) divided the total cohesive energy density (δ_total_) from Hildebrand's theory into three aspects in terms of atomic dispersion forces (δ_d_), molecular polar forces from dipole moments (δ_p_) and intermolecular hydrogen-bonds (δ_h_). The equation for HSP is as follows,

(14) $${\delta_{\mathrm{total}}}^{2}={\delta_{d}}^{2}+{\delta_{p}}^{2}+{\delta_{h}}^{2}$$

where δ_total_ is Hansen total solubility parameter, which now consists of three solubility parameters in terms of dispersion (δ_d_), polar (δ_p_) and hydrogen-bonding (δ_h_).

The theoretical miscibility between CO and solvents could be studied by their HSP predictions through Eq. (15), (16) (Li et al., 2014), where simplified molecular input line entry syntax (SMILES) notations of both solvents and solute were transformed to predict physicochemical properties (e.g., Log P and boiling point, etc.) and 3-D HSP sphere with corresponding relative energy difference (RED) values in the HISPIP software (Version 6.0.04, Denmark). In general, the RED value ranging from 0 to 1 could indicate the affinity between solvents and solutes, which could provide a rapid and useful way to screen good and bad solvents for specific target solutes.

(15) $${R_{a}}^{2}=4{\left({\delta_{d}^{\mathrm{solvent}}}^{2}-{\delta_{d}^{\mathrm{solute}}}^{2}\right)}^{2}+{\left({\delta_{p}^{\mathrm{solvent}}}^{2}-{\delta_{p}^{\mathrm{solute}}}^{2}\right)}^{2}+{\left({\delta_{h}^{\mathrm{solvent}}}^{2}-{\delta_{h}^{\mathrm{solute}}}^{2}\right)}^{2}\;$$

(16) $$\text{Relative Energy Difference}\;\left(\mathrm{RED}\right)=\frac{R_{a}}{R_{0}}\;$$

where R_a_ represents the distance of the solvent and solute and R_0_ represents the radius of the solubility model.

### 2.10 Hurdle technology for solvent screening

**Fig. 1:**
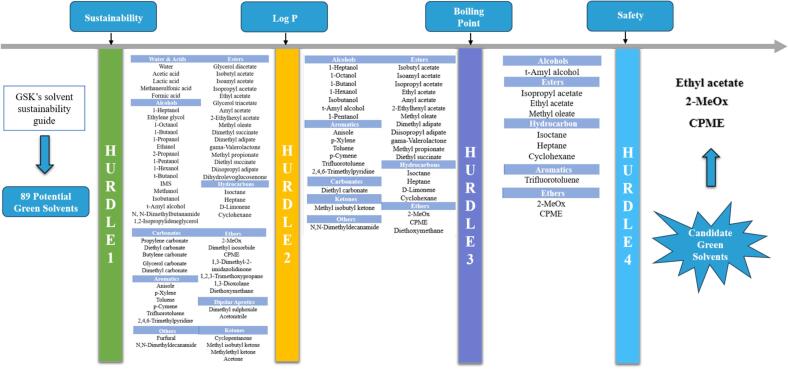
Hurdle technology for solvent screening.

The hurdle concept has become a promising decision-making tool for solvent screening once the factual hurdles are appropriately established (Khan et al., 2017). As Fig. 1 illustrates, a list of candidate solvents was collected from the GlaxoSmithKline (GSK)’s solvent sustainability guide based on their color assignments (Alder et al., 2016), where solvents with green and amber colors were screened out for further hurdle factors associated with technical properties like Log P and boiling point, and safety considerations. The solvents screened from each hurdle factor are consolidated in Supplementary materials, which helps to make an informed decision based on the theoretical data predicted.

### 2.11 Statistical analysis

All experiments were repeated in triplicate and the data were expressed as means ± standard deviations. The statistical analysis of variance (ANOVA) was determined by the SPSS Statistics 16.0 software (IBM Corp., Armonk, NY, USA) using the Duncan's test with a significant difference at 95 % level between samples. Significant differences (*p* < 0.05) showed by each sample tested were labeled as different superscript letters.

## 3 Results and discussion

### 3.1 Solvent screening based on theoretical studies

Based on the GSK's solvent sustainability guide, this study systematically screened green solvents using hurdle technology to address challenges in the CO extraction process. Sixty-seven solvents with green or amber composite color were firstly selected as potential green solvents from the GSK solvent library with an overall sustainable assessment including waste, environmental, health, safety, and life cycle assessment scores. These solvents were further screened by Log P as the 2nd hurdle, where 35 solvents were selected related to their lipid dissolving capacities. Log P is the log of the partition coefficient of a solute between octanol and water (i.e., two non-miscible solvents) at near infinite dilution (Datta et al., 2021), which as widely used to estimate the lipophilicity of a molecule. The lower Log *P* value of solvents indicated their hydrophilicity while solvents with a relatively high Log P value reveal their lipophilicity. As shown in Supplementary Table 1, solvents with higher Log *P* values (in green) are more effective for dissolving oils, followed by medium (in yellow) and poor (in red) lipid dissolving capacities. Considering the energy consumption related to solvent recovery, boiling point was used as the 3rd hurdle. Since solvents with high boiling points (in red) are difficult to remove while those with excessively low boiling points are challenging to store and extract, solvents in green with medium boiling points (70–106 °C) were preferential (Supplementary Table 2). In the final hurdle, ten solvents were further screened regarding to their safety concerns including toxicity index to human and environment, and flammability or explosivity as well. Hence, three green solvents including ethyl acetate, 2-MeOx, and CPME stood out as candidate alternatives to n-hexane for the following experiments.

### 3.2 The effect of alternative solvents on the CO extraction

**Fig. 2:**
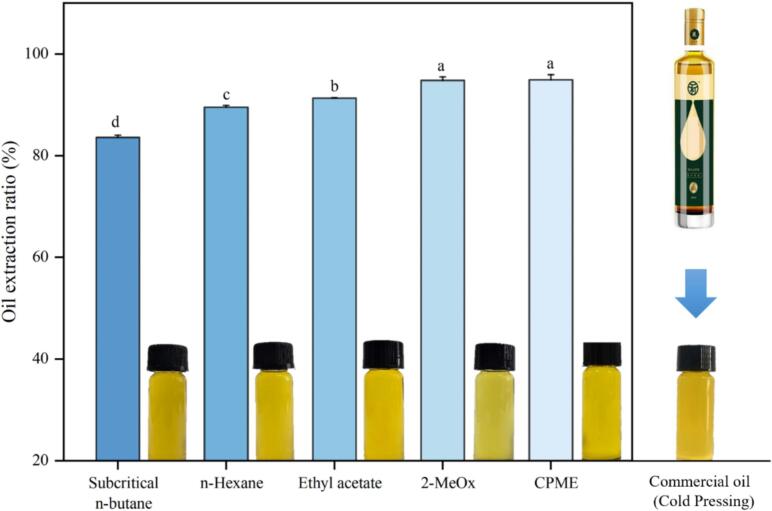
The effect of different solvents on the extraction ratio of lipid extracted from Camellia seed oil cakes.

The initial moisture and oil content in camellia seed cakes were determined to as 3.78 % ± 0.00 and 9.35 % ± 0.00, respectively. The effect of different solvents on the extraction ratio of CO is illustrated in Fig. 2. It was interesting to notice that subcritical butane performed the worst (83.75 % ± 0.43) among all solvents. This could be explained by excessive solvent volatilization and retrograde solubility of lipids in subcritical butane as the solvent density decreased with temperature increased above 55 °C (Chen et al., 2023). Similarly, the single extraction efficiency of subcritical n-butane (78.01 %) was the lowest for acer truncatum seed oil compared to its twice extraction (94.50 %) and ultrasound-assisted n-hexane extraction (86.04 %) (Wang et al., 2023). The extraction ratio of n-hexane was lower (89.50 % ± 0.00) than selected green solvents, where 2-MeOx (94.79 % ± 0.00) and CPME (94.91 % ± 0.00) showed similar extraction ratios, followed by ethyl acetate (91.30 % ± 0.00). Furthermore, it is noteworthy that all solvent extracted COs presented a transparent golden yellow appearance. Compared to commercial cold pressed CO, subcritical n-butane extracted CO with a slightly dark color whereas 2-MeOx could obtain more desirable CO with bright gold color.

As Table 1 shown, regardless of the solvent used, a similar composition was found in different CO extracted in terms of acylglycerols and fatty acids. Firstly, the effect of subcritical n-butane on the composition of acylglycerols and fatty acids was significantly different from other solvents, where the content of free fatty acids (FFA) was the highest corresponding to the highest AV, but the content of monounsaturated fatty acids (MUFA) was the lowest accompanying with highest level of saturated fatty acids (SFA) and polyunsaturated fatty acids (PUFA). CO was demonstrated to have more than 96 % of triacylglycerols (TAGs), in which oleic acid was characterized as the predominant fatty acid, followed by linoleic and palmitic acids, as previously mentioned (Rodrigues et al., 2021; Shi et al., 2020). In addition to subcritical n-butane, all other solvents presented a comparable acylglycerol and fatty acid composition to commercial cold pressed oil. The performance of 2-MeOx showed significant selectivity in extracting myristic acid compared to other solvents, resulting in a relatively balanced fatty acid composition. Considering the results above, solvent extraction could obtain the CO with similar acylglycerol and fatty acid compositions to commercial cold pressed CO, where the performance of alternative solvents (e.g., 2-MeOx, CPME and ethyl acetate) outweighed conventional n-hexane and subcritical n-butane due to their high yields without significant changes in acylglycerol and fatty acid compositions, which could be applied for further investigations.

**Table 1:** Acylglycerol and fatty acid composition, and basic physicochemical properties of lipids extracted from Camellia seed oil cakes by different solvents.

	Subcritical Butane	Commercial oil (Cold Pressing)	n-Hexane	Ethyl acetate	2-methyloxolane (2-MeOx)	Cyclopentyl methyl ether (CPME)
**Acylglycerols (%)**						
Free fatty acids	2.15 ± 0.11^a^	1.12 ± 0.05^b^	1.24 ± 0.12^b^	1.24 ± 0.02^b^	1.21 ± 0.17^b^	1.24 ± 0.02^b^
Monoacylglycerol	–	0.04 ± 0.01^b^	0.05 ± 0.01^b^	0.05 ± 0.00^b^	0.04 ± 0.01^b^	0.06 ± 0.01^b^
Diacylglycerol	1.11 ± 0.22^c^	1.31 ± 0.21^b^	1.67 ± 0.21^a^	1.49 ± 0.04^a, b^	1.57 ± 0.01^a^	1.48 ± 0.05^a, b^
Triacylglycerol	96.75 ± 0.12^d^	97.53 ± 0.25^a^	97.05 ± 0.06^b, c^	97.25 ± 0.05^b^	97.18 ± 0.18^b, c^	97.24 ± 0.06^b^

**Fatty acids (%)**						
Myristic acid (C14:0)		–	–	–	1.65 ± 0.17	–
Palmitic acid (C16:0)	10.07 ± 1.06^a^	6.53 ± 0.09^b^	7.09 ± 0.71^b^	6.86 ± 0.06^b^	6.89 ± 0.17^b^	7.07 ± 0.09^b^
Stearic acid (C18:0)	1.87 ± 0.07^a^	1.45 ± 0.10^b^	1.47 ± 0.17^b^	1.40 ± 0.07^b^	1.27 ± 0.01^c^	1.40 ± 0.02^b^
Oleic acid (C18:1)	77.04 ± 2.25^c^	83.37 ± 0.10^a^	83.23 ± 0.08^a^	82.91 ± 0.06^a, b^	81.38 ± 0.23^b^	82.67 ± 0.00 ^a, b^
Linoleic acid (C18:2)	10.24 ± 1.23^a^	8.03 ± 0.08^b^	7.88 ± 0.47^b^	8.14 ± 0.11 ^b^	8.14 ± 0.10^b^	8.20 ± 0.12^b^
Linolenic acid (C18:3)	0.77 ± 0.11^a^	0.63 ± 0.01^b^	0.63 ± 0.08^b^	0.70 ± 0.04^a, b^	0.67 ± 0.03^a, b^	0.68 ± 0.01^a, b^
∑SFA	11.94 ± 1.14^a^	7.97 ± 0.20^c^	8.56 ± 0.88^c^	8.26 ± 0.13^c^	9.81 ± 0.1^b^	8.46 ± 0.11^c^
∑MUFA	77.04 ± 2.25^c^	83.37 ± 0.10^a^	83.23 ± 0.08^a^	82.91 ± 0.06^a, b^	81.38 ± 0.2^b^	82.67 ± 0.00^a, b^
∑PUFA	11.02 ± 1.11^a^	8.66 ± 0.10^b^	8.51 ± 0.56^b^	8.84 ± 0.06^b^	8.81 ± 0.13^b^	8.88 ± 0.11^b^
∑UFA	88.06 ± 1.14^c^	92.03 ± 0.20^a^	91.73 ± 0.48^a^	91.75 ± 0.12^a^	90.19 ± 0.10^b^	91.55 ± 0.11^a^
Acid value (mg/g)	0.24 ± 0.03^a^	0.04 ± 0.01^e^	0.09 ± 0.02^d^	0.12 ± 0.02^b, c^	0.14 ± 0.02^b^	0.12 ± 0.02^b, c^
Peroxide value (g/100 g)	0.11 ± 0.00^b^	0.05 ± 0.01^f^	0.03 ± 0.00^d. e^	0.08 ± 0.01^c^	0.17 ± 0.01^a^	0.06 ± 0.01^d^
Oxidation onset temperature (°C)	206.52 ± 0.07^b^	205.59 ± 0.49^b,c^	206.09 ± 0.91^b, c^	203.59 ± 0.04^d^	209.26 ± 0.37^a^	205.22 ± 0.06^c^

SFAs: saturated fatty acids, MUFAs: monounsaturated fatty acids, PUFAs: polyunsaturated fatty acids, UFAs: unsaturated fatty acids.

Values followed by the same letter are not significantly at p < 0.05, which are presented as mean ± standard deviation of triplicate.

### 3.3 Analysis of CO extracted by different solvents

#### 3.3.1 Physicochemical properties

As can be seen in Table 1, the acid and peroxide values of all oil samples are in accord with the national standard though significant differences are found between them. Compared to commercial camellia oil, the AV of solvent extracted oils is relatively higher due to the higher content of FFA or phenolic compounds. The relatively low peroxide value indicates the low degree of lipid oxidation in CO, which may be attributed to the presence of minor compounds with antioxidant activities (Qin et al., 2020). The POV of oils extracted by n-hexane, ethyl acetate and CPME is comparable to that of commercial camellia oil while oils from subcritical n-butane and 2-MeOx extraction has relatively higher POV.

The oxidative stability of plant oils depends on many factors, including lipid composition and trace elements (Bayram & Decker, 2023; Brahim & Bouaziz, 2019), especially for the unsaturated degree of fatty acids, and the level of endogenous antioxidants like tocopherols and phenolic compounds. The oxidative stability in Table 1 showed that the OOT of all solvent-extracted oils was comparable to commercial CO, where 2-MeOx performed the best oxidative stability, followed by subcritical n-butane, n-hexane, CPME, and ethyl acetate. This may be due to the influence of both unsaturation level and micronutrient composition in oil samples.

#### 3.3.2 Lipid concomitant analysis

**Fig. 3:**
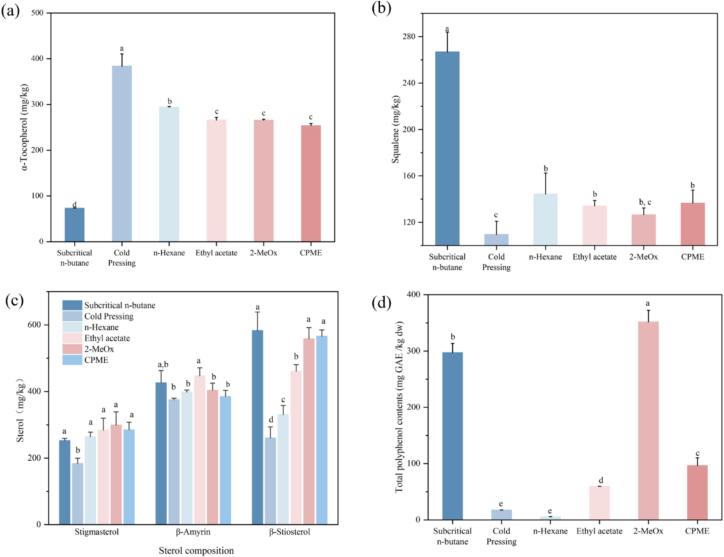
The effect of different solvents on the lipid concomitants in oils extracted from Camellia seed oil cakes: (a) α-Tocopherol, (b) Squalene, (c) Sterol, (d) Total phenolic content.

Apart from triacylglycerols as major components in CO, there are also some minor components like tocopherol, squalene, sterol and phenolic compounds that could be recognized as interesting lipid concomitants due to their favorable bioactivities and health effects (Wu et al., 2020). As illustrated in Fig. 3, commercial camellia oil has the lowest levels of squalene, sterols, and TPC though it has the highest α-tocopherol content (383.74 ± 26.70 mg/kg), which is above the average (153–771 mg/kg) in Chinese camellia oils (Gao et al., 2024). Such tocopherol could enhance oxidative stability of bulk edible oils above 20 ppm (Mishra et al., 2021). Interestingly, subcritical n-butane nearly has the opposing situation compared to this, where the content of all lipid concomitants could be significantly enhanced except for heat-sensitive α-tocopherol. It is worth mentioning that three candidate green solvents could extract more sterols and TPC than n-hexane whereas n-hexane could obtain a slightly higher content of α-tocopherol and squalene than green solvents. The phytosterol content in CO extracted by green solvent could be remarkably enhanced, especially for the presence of unique 4,4-dimethyl phytosterol like beta-amyrin (Zhang et al., 2020). This triterpene alcohol has drawn increasing attentions because of its considerable anti-inflammatory effect (Li et al., 2025; Nogueira et al., 2019). Phenolic extracts from plants or agro-industrial by-products have been recently investigated as new natural antioxidant to improve quality and shelf life of vegetable oils and other foods (Sordini et al., 2024). The highest TPC (351.6 4 ± 20.72 mg GAE/kg dw) could be obtained by 2-MeOx due to its better water miscibility during extraction, which was much higher than that in most extra virgin olive oils of different cultivars (Korkmaz, 2023; Olmo-Cunillera et al., 2024). This TPC level is much higher than normal cold-pressed camellia oil (Gao et al., 2024), which might lead to the higher antioxidant activity corresponding to the best oxidative stability previously mentioned.

### 3.4 Kinetics study and energy consumption for alternative solvent extractions

**Fig. 4:**
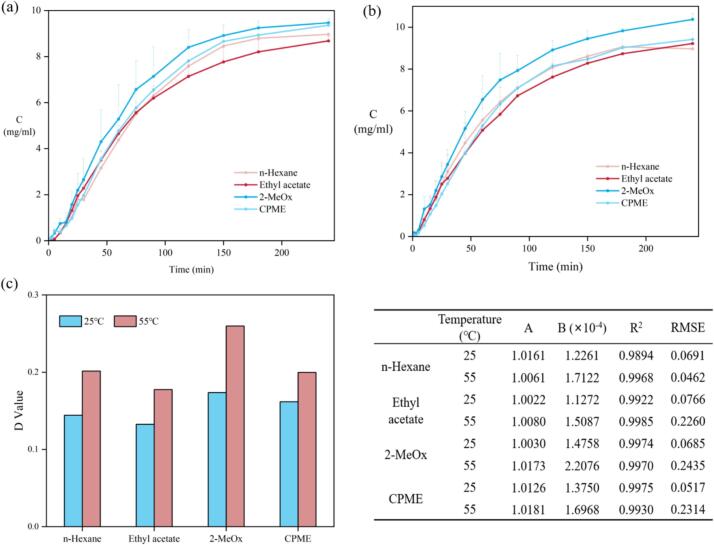
kinetics study of lipid extraction from Camellia seed oil cakes using candidate green solvents and n-hexane. (For interpretation of the references to color in this figure legend, the reader is referred to the web version of this article.)

After evaluation of CO’ quality, it is crucial to assess the feasibility of replacing hexane with green solvents at an industrial scale. Extraction kinetics are particularly important as they directly affect residence times inside the extractors, thus affecting the productivity of plants. In this experiment, the mass transfer processes of different solvents at 25 °C and 55 °C were measured. The model derived from Fick's law is usually used to model the solid-liquid extraction process for a better explanation of the extraction phenomenon, where the smaller RMSE value indicates the smaller deviation between predicted and actual values. As shown in Fig. 4, the RMSE value of all solvents at different temperatures is less than 0.1 while their correlation coefficients R^2^ are very close to 1, indicating the high degree of fitting for the model corresponding to the CO extraction. The model constant A is thought to be associated with a relevant washing stage, and the diffusion rate constant B is proven to be related to temperature and solvent (Baümler et al., 2016). Fig. 4 shows the extraction process of each solvent at different temperatures, where the B value of 2-MeOx is the highest at both temperatures, indicating the most efficient extraction. Although the properties of biomaterials such as porosity and density affect the diffusion of oils from feedstock (Naik et al., 2021), the D_e_ value as the effective diffusion coefficient is solely related to temperature (Chan et al., 2014). The temperature increase could indeed achieve a more effective diffusivity for all solvents, where 2-MeOx always exhibited the highest diffusion rate at both 25 °C and 55 °C, indicating its superiority as an alternative solvent. Therefore, 25 °C is considered the optimal temperature for the extraction process with the consideration of both extraction efficiency and operating cost for industrial-scale production.

The environmental impact of different solvents was evaluated by comparing the energy consumption and CO_2_ emissions associated with CO extraction and solvent recovery. As Supplementary Table 3 presented, the energy consumption of all solvents for the extraction process only is comparable excepting for subcritical n-butane extraction, which integrates solvent recovery together. However, the higher energy consumption for solvent recovery process is obtained by 2-MeOx and CPME due to their higher boiling points and enthalpies of vaporization. However, it is clear superiority for bio-based 2-MeOx derived from renewable resources in terms of environment and safety (Morón-Ortiz et al., 2024). As previously reported, 800 g of CO_2_ will be released into the atmosphere by the combustion of fossil fuels during the process of generating 1kWh of electricity (Qiu et al., 2022). Therefore, no significant difference was found for total CO_2_ emission except CPME, where subcritical n-butane extraction showed the lowest CO_2_ emission (0.19 ± 0.00 kg of CO_2_ emission) while CPME extraction released 0.39 ± 0.12 kg of CO_2_ compared to n-hexane (0.23 ± 0.04 kg of CO_2_ emission).

In addition to extraction efficiency (i.e., extraction yield, solvent power and selectivity) and environmental impact (i.e., energy requirements) of green extraction solvents, it is also important to look at their economic feasibility and scalability for a thorough performance evaluation (Usman et al., 2023). For alternative green solvents, the cost-effectiveness of extracting more desired lipid concomitants in a more effective extraction process could cut down on expenses. Moreover, these solvents are recovered and reused for cost savings in the long run, which could be available for scaling up with the consideration of raw materials, extraction process and facility, and energy needs. Hence, the environmental, productive potential and operation benefits from alternative green solvents are more favorable from the perspective of long-term sustainability.

### 3.5 Theoretical solubility of oil constituents extracted in solvents

The theoretical miscibility between solvents and solutes could be predicted base on their chemical structures using in HSPiP software of the newest version. Although some solvents and solutes were nonexistent in the embedded HSP database, the Yamamoto Molecule break (Y-MB) method could break SMILES into functional groups for HSP prediction and further estimations of miscibility through RED values. For instance, the chemical structure of major triglyceride in CO comprising three oleic acids could be transformed to its canonical SMILES notation as CCCCCCCC/C=C\CCCCCCCC(=O)OCC(OC(=O)CCCCCCC/C=C\CCCCCCCC)COC(=O)CCCCCCC/C=C\CCCCCCCC for predicting HSP. Similarly, the HSP of triglyceride with two oleic acid and one linoleic acid chains, as well as α-tocopherol, sterols and squalene, was used in the Hansen solubility modeling with RED value as the criterion for evaluating theoretical miscibility. In the HSP modeling, the solvent with a RED number ≤ 1 could be generally considered to have good affinity to the target solute whereas the solvent with a RED number > 1 has a relatively bad dissolving capability. In order to distinguish the difference of dissolving power between solvents with defined good miscibility, RED number ≤ 1 was further divided into two groups, where 0.6 ≤ RED ≤1 represented medium miscibility between solvents and solutes while 0 < RED <0.6 was indicated as relatively good miscibility.

As shown in Table 2, CPME and 2-MeOx with relatively small RED values presented the better theoretical miscibility with all solutes in CO, followed by n-hexane, ethyl acetate and subcritical n-butane. These predictions basically coincide with experimental results, which further proved that candidate green solvents could be preferential solvents for the extraction of oils from LC kernels as compared to n-hexane and subcritical n-butane. It is also important to note that the theoretical miscibility is based on thermodynamics rather than kinetics. Although this case-by-case study have divergences with experimental results, the HSP prediction could still applied as a heuristic tool for better understanding the dissolving mechanism underlying lipid extractions.

**Table 2:** Theoretical miscibility between oil components as solutes and solvents based on the relative energy difference (RED) values predicted by their Hansen solubility parameters.

	δ_d_	δ_p_	δ_H_	Subcritical n-butane	n-Hexane	Ethyl acetate	2-MeOx	CPME
TAG_1_	16.5	1.5	2.6	0.82	0.55	0.77	0.50	0.41
TAG_2_	16.5	1.6	3.1	0.85	0.59	0.71	0.46	0.37
α-Tocopherol	16.9	1.5	2.6	0.96	0.70	0.71	0.46	0.36
Squalene	17.0	0.9	3.1	0.94	0.66	0.81	0.56	0.46
Stigmasterol	17.4	1.8	3.6	1.08	0.80	0.74	0.48	0.37
β-Sitosterol	17.2	1.8	3.4	1.02	0.75	0.73	0.46	0.35
β-Amyrin	17.7	1.7	2.9	1.11	0.85	0.82	0.56	0.45

RED >1: Bad miscibility 0.6 ≤ RED ≤1: Medium miscibility 0 < RED <0.6: Good miscibility.

TAG_1_: Triglyceride (R_1_:C18:1, R_2_: C18:1, R_3_: C18:1).

TAG_2_: Triglyceride (R_1_:C18:2, R_2_: C18:1, R_3_: C18:1).

## 4 Conclusion

The effect of petroleum-based and alternative green solvents on the lipid extraction from Camellias seed oil cakes was originally investigated in this study. Green solvents proved to be superior to petroleum-based solvents in both theoretical and experimental results for an efficient green extraction of CO considering higher yield, similar acylglycerol and fatty acid composition, and more abundant lipid concomitants. The CO extracted by green solvents could obtain more squalene, sterol and phenolic compounds than commercial cold pressing oil. Although there is a significant difference in physicochemical properties of solvent extracted COs, their basic oil quality indices are still within the occupation standard range. Moreover, 2-MeOx with better water miscibility could obtain CO with the best oxidative stability because of its highest TPC level after extraction. Besides, the predicted HSP helped to explain the theoretical miscibility between solvents and oil components, which further demonstrates the dissolving mechanism behind CO extractions from the aspect of thermodynamics. Although CPME showed comparable solvent power to 2-MeOx in the CO extraction, the bio-based 2-MeOx seems more sustainable from the practical point of view. From the results above, 2-MeOx exhibits its unique advantages in CO extraction compared to other solvents and commercial cold pressed CO, which deserves to be further explored for its process intensification and applicability to other oilseeds.

## CRediT authorship contribution statement

**Yingyi Lin:** Writing – original draft, Visualization, Methodology, Investigation, Formal analysis, Data curation. **Yong Wang:** Supervision, Resources. **Ying Li:** Writing – review & editing, Validation, Supervision, Software, Project administration, Methodology, Investigation, Funding acquisition, Formal analysis, Data curation.

## Declaration of competing interest

The authors declare that they have no known competing financial interests or personal relationships that could have appeared to influence the work reported in this paper.

## Data Availability

Data will be made available on request.

## Appendix A Supplementary data

Supplementary material: Table 1: Log P value as the second hurdle for solvent screening. Table 2: Boiling point as the thrid hurdle for solvent screening. Table 3: Energy consumption and CO_2_ emission for different solvent extraction of lipids from Camellia seed oil cakes. Image 1
