# Supplementary material for: Exploring alternative solvents to n-hexane for green extraction of lipid from camellia oil cakes
Source: Food Chem X. 2025 Apr 5;27:102443. doi: 10.1016/j.fochx.2025.102443 (PMC12005931; doi:10.1016/j.fochx.2025.102443)
Supplement: Supplementary file 1 — Supplementary material: Table 1: Log P value as the second hurdle for solvent screening. Table 2: Boiling point as the thrid hurdle for solvent screening. Table 3: Energy consumption and CO2 emission for different solvent extraction of lipids from Camellia seed oil cakes. [file mmc1.docx]

**Supplementary materials**

Table 1: Log P value as the second hurdle for solvent screening

Table 2: Boiling point as the thrid hurdle for solvent screening

Table 3: Energy consumption and CO_2_ emission for different solvent extraction of lipids from Camellia seed oil cakes.

**Table 1.**

|  | Solvent | Log P |  | Solvent | Log P |
| --- | --- | --- | --- | --- | --- |
| Water & Acids | Water | -0.5 | Carbonates | Propylene carbonate | -0.4 |
|  | Acetic acid | -0.2 |  | Diethyl carbonate | 1.2 |
|  | Lactic acid | -0.7 |  | Butylene carbonate | 0.1 |
|  | Methanesulfonic acid | -0.9 |  | glycerol carbonate | -1.5 |
|  | Formic acid | -0.2 |  | Dimethyl carbonate | 0.5 |
| Alcohols | 1-Heptanol | 2.7 | Esters | Glycerol diacetate | -0.3 |
|  | Ethylene glycol | -1.4 |  | Isobutyl acetate | 1.8 |
|  | 1-Octanol | 3.0 |  | Ethyl acetate | 0.7 |
|  | 1-Butanol | 0.9 |  | Isoamyl acetate | 2.0 |
|  | 1-Propanol | 0.3 |  | Glycerol triacetate | 0.2 |
|  | Ethanol | 0.3 |  | Amyl acetate | 1.9 |
|  | 2-Propanol | 0.3 |  | 2-Ethylhexyl acetate | 3.2 |
|  | t-butanol | 0.5 |  | Isopropyl acetate | 0.9 |
|  | IMS | -0.1 |  | Dihydrolevoglucosenone | -0.2 |
|  | Methanol | -0.3 |  | Dimethyl adipate | 1 |
|  | 1-Pentanol | 1.6 |  | Methyl oleate | 7.6 |
|  | 1-Hexanol | 2.0 |  | Dimethyl succinate | 0.4 |
|  | 1,2-Isopropylideneglycerol | -0.2 |  | Dimethyl adipate | 1.0 |
|  | N, N-Dimethylbutanamide | 0.6 |  | Diethyl succinate | 1.2 |
|  | t-Amyl alcohol | 0.9 |  | Diisopropyl adipate | 2.2 |
|  | Isobutanol | 0.8 |  | gama-Valerolactone | 0.8 |
| Hydrocarbons | Heptane | 4.4 | Ketones | Cyclopentanone | 0.4 |
|  | Cyclohexane | 3.4 |  | Methyl isobutyl ketone | 1.3 |
|  | D-Limonene | 2.44 |  | methylethyl ketone | 0.3 |
|  | Isoctane | 3.8 |  | acetone | -0.1 |
| Others | Furfural | 0.4 | Dipolar Aprotics | Dimethyl sulphoxide | -0.6 |
|  | N, N-Dimethyldecanamide | 4.0 |  | Acetonitrile | 0 |
| Aromatics | Anisole | 2.1 | Ethers | Dimethyl isosorbide | -0.6 |
|  | 2,4,6-Trimethylpyridine | 1.9 |  | CPME | 1.3 |
|  | p-Xylene | 3.2 |  | 2-MeOx | 1 |
|  | Toluene | 2.7 |  | 1,3-Dimethyl-2-imidazolidinone | -0.5 |
|  | Trifluorotoluene | 3 |  | 1,2,3-Trimethoxypropane | -0.1 |
|  | p-Cymene | 4.1 |  | 1,3-Dioxolane | -0.4 |
|  |  |  |  | Diethoxymethane | 0.8 |

LogP < 0 0 ≤ RED < 0.7 RED ≥ 0.7

**Table 2.**

|  | Solvent | Boiling point |  | Solvent | Boiling point |
| --- | --- | --- | --- | --- | --- |
| Alcohols | 1-Heptanol | 178 | Esters | Isobutyl acetate | 116 |
|  | 1-Octanol | 195 |  | Isoamyl acetate | 142 |
|  | 1-Butanol | 118 |  | Amyl acetate | 146 |
|  | Isobutanol | 108 |  | 2-Ethylhexyl acetate | 199 |
|  | 1-Hexanol | 157 |  | Methyl oleate | 218 |
|  | t-Amyl alcohol | 102 |  | Diethyl succinate | 218 |
|  | 1-Pentanol | 137 |  | Dimethyl adipate | 136 |
| Aromatics | p-Xylene | 138 |  | Diisopropyl adipate | 405 |
|  | Toluene | 111 |  | Isopropyl acetate | 89 |
|  | Trifluorotoluene | 102 |  | Ethyl acetate | 77 |
|  | 2,4,6-Trimethylpyridine | 171-172 |  | gama-Valerolactone | 207 |
|  | Anisole | 154 |  | Methyl propionate | 79 |
|  | p-Cymene | 177 | Others | N, N-Dimethyldecanamide | 110~111 |
| Hydrocarbons | Heptane | 98 | Ethers | CPME | 106 |
|  | Cyclohexane | 81 |  | 2-MeOx | 78 |
|  | D-Limonene | 175 |  | Diethoxymethane | 42 |
|  | Isoctane | 99 | Ketones | Methyl isobutyl ketone | 117 |
| Carbonates | Diethyl carbonate | 126 |  |  |  |

Boiling point < 70℃ or >120℃ 106℃ ≤ Boiling point≤ 120℃ 70℃ **<** Boiling point < 106℃

**Table 3.**

| Process |  | Subcritical n-butane | n-Hexane | Ethyl acetate | 2-MeOx | CPME |
| --- | --- | --- | --- | --- | --- | --- |
| Extraction | PC (kWh) | 0.24±0.00^a^ | 0.02±0.01^c^ | 0.02±0.01^c^ | 0.05±0.03^b^ | 0.02±0.00^c^ |
|  | CO_2_ (kg) | 0.19±0.00^a^ | 0.01±0.00^c^ | 0.02±0.08^c^ | 0.04±0.02^b^ | 0.02±0.00^c^ |
| Solvent evaporation | PC (kWh) | - | 0.27±0.06^b^ | 0.32±0.06^a, b^ | 0.43±0.05^a, b^ | 0.47±0.16^a^ |
|  | CO_2_ (kg) | - | 0.22±0.04^b^ | 0.26±0.05^a, b^ | 0.34±0.04^a, b^ | 0.38±0.12^a^ |
| Total energy consumption | PC (kWh) | 0.24±0.00^b^ | 0.29±0.03^b^ | 0.34±0.06^a,b^ | 0.48±0.09^a^ | 0.49±0.16^a^ |
|  | CO_2_ (kg) | 0.19±0.00^b^ | 0.23±0.04^b^ | 0.27±0.05^b^ | 0.38±0.07^b^ | 0.39±0.12^a^ |
